# Transcallosal Inhibition during Motor Imagery: Analysis of a Neural Mass Model

**DOI:** 10.3389/fncom.2017.00057

**Published:** 2017-06-30

**Authors:** Anna L. Mangia, Mauro Ursino, Maurizio Lannocca, Angelo Cappello

**Affiliations:** Department of Electrical, Electronic and Information Engineering, University of BolognaCesena, Italy

**Keywords:** ERD/ERS, EEG, neural mass model, transcallosal connection, Theory of Inhibition, Theory of Excitation

## Abstract

The EEG rhythmic activities of the somato-sensory cortex reveal event-related desynchronization (ERD) or event-related synchronization (ERS) in beta band (14–30 Hz) as subjects perform certain tasks or react to specific stimuli. Data reported for imagination of movement support the hypothesis that activation of one sensorimotor area (SMA) can be accompanied by deactivation of the other. In order to improve our understanding of beta ERD/ERS generation, two neural mass models (NMM) of a cortical column taken from Wendling et al. (2002) were interconnected to simulate the transmission of information from one cortex to the other. The results show that the excitation of one cortex leads to inhibition of the other and vice versa, enforcing the Theory of Inhibition. This behavior strongly depends on the initial working point (WP) of the neural populations (between the linear and the upper saturation region of a sigmoidal function) and on how the cortical activation or deactivation can move the WP in the upper saturation region ERD or in the linear region ERS, respectively.

## Introduction

An important feature of the brain is its ability to generate characteristic rhythms in its activity. The frequency of such brain oscillations depends both on the membrane properties of the single neurons and on the organization and interconnectivity of networks to which they belong (da Silva, 1991).

The EEG rhythmic activities of the somato-sensory cortex (Sensory Motor Rhythms, SMR) are commonly modulated when subjects perform certain tasks or react to specific stimuli. Generally, it is assumed that the spectra band power is related to the degree of synchrony of the underlying oscillating neuronal population. In particular, a decrease in mu (8–13 Hz) or beta (14–30 Hz) rhythmic activity occurring after a given event, which manifests as a decrease in the spectra band power, is called event-related desynchronization (ERD), whereas the inverse is called event-related synchronization (ERS). Both mu and beta rhythm ERD/ERS patterns are associated with real and imagined movement, and these features are much used for brain computer interface (BCI) control.

Different mechanisms are probably at the origin of these two rhythms. Hughes and Crunelli (2005) discovered that the occurrence of mu oscillations depends on the activity of a subset of thalamocortical neurons (Hughes and Crunelli, 2005). Conversely, several experiments found out that oscillations in the beta frequency range are easily detectable in different cortical sites, but not in simultaneously obtained recordings from thalamic electrodes. These findings have led to the interpretation that these rhythmic activities are primarily generated in the cortex itself (da Silva, 2010).

Pfurtscheller and Neuper studied the spatiotemporal patterns of mu and beta rhythms during motor imagery with a dense array of EEG electrodes. The subjects were instructed to imagine movements of either the right or the left hand. These rhythms displayed an ERD only over the contralateral hemisphere, while an enhancement of the rhythms ERS was found over the ipsilateral side. Furthermore, Pfurtscheller (2006), using a qualitative model, proposed that ERD or ERS, induced by a sudden change of activation, may depend on the initial working point (WP). At certain physiological levels of activation, an increase in activation results in ERD, whereas activation decrease results in ERS.

The previous data support the hypothesis that activation of one sensorimotor area (SMA) can be accompanied by deactivation of the other (Pfurtscheller and Neuper, 1997). The ipsilateral synchronization can be interpreted as deactivation or active inhibition of the ipsilateral sensorimotor structures. It is not surprising that, for an optimal performance of a specific task (e.g., imagination of the right hand movement), cortical areas not specifically involved in the task may be deactivated or inhibited for information processing (Pfurtscheller, 1992; Pfurtscheller and Neuper, 1997). By way of example, enhanced inhibition of the homotrophic motor area via the transcallosal fiber system, was reported by Netz et al. (1995).

The corpus callosum is a brain structure that connects the left and the right cerebral hemispheres. Intrahemispheric communication occurs by means of axonal projections connecting cortexes both with cortico-cortical and cortico-subcortical pathways. It is however still uncertain how the corpus callosum regulates transfer and communication between hemispheres, as studies investigating its role report conflicting statements.

Some studies suggest that the corpus callosum could play an inhibitory role (Theory of Inhibition), whereas others say that the corpus callosum serves an excitatory function (Theory of Excitation) (Clarke and Zaidel, 1994; Bloom and Hynd, 2005). The inhibitory model poses that the corpus callosum maintains independent processing between the two hemispheres, hindering activity in the opposing hemisphere and causing greater connectivity to increase lateralization (positively correlated) (Welcome and Chiarello, 2008; Adam and Güntürkün, 2009). Lateralization of the brain hemispheres refers to a functional dominance of one hemisphere over the other, in which one is more responsible or entirely responsible for control of a function in comparison to the other (Noggle and Hall, 2011). The excitatory model poses that the corpus callosum shares and integrates information between hemispheres, causing greater connectivity to decrease laterality effects by masking the underlying hemispheric differences in tasks that require interhemispheric exchange (negatively correlated) (Clarke and Zaidel, 1994; Bloom and Hynd, 2005; van der Knaap and van der Ham, 2011).

A deeper understanding of the EEG signal and of the neurophysiological information it contains can be gained through the use of biologically inspired neurocomputational models. In particular, two main classes of models have been proposed to simulate cortical rhythms: models with spiking neurons (see Dayan and Abbott, 2001 for a review) and neural mass models (NMM). In the latter, the main variables represent the cumulative activity of population of neurons (instead of single cells) which share a similar membrane potential and exhibit the same dynamical behavior. Among the others, NMMs have been successfully used to simulate specific aspects of electrical brain activity, such as alpha rhythms (Jansen and Rit, 1995), oscillations and synchronization in the γ-band (Schillen and König, 1994), dynamics in the olfactory cortex (Freeman, 1987), epileptic patterns (Wendling et al., 2002) and brain rhythms during sleep (Cona et al., 2014).

In order to test the phenomenon of mu ERD/ERS generation, Suffczynsky et al. used a computational model of interacting neural populations, with the emphasis on thalamo-cortical networks (Suffczynski et al., 2001). Even though intra-cortical networks also play an important role in rhythms propagation, the model in Suffczynski et al. (2001) does not include these, rather focusing on the system responsible for the generation and modulation of mu rhythmic activities, namely the thalamo-cortical circuit. However, this simplification cannot be valid for beta ERD/ERS, because of their origin in the cortex. Pfurtscheller proposed a cortical activation model to explain whether an internally or externally paced event induces an ERD or ERS in a specific frequency band (Pfurtscheller, 2006). He found that, depending on the baseline level of cortical activation, a sudden change in input can induce either ERD or ERS in a given area. This study introduces the concept of WP as the level of activation from which different behaviors can be obtained. In particular, when the baseline of cortical activation is low and most of the neurons in a given area are still available for synchronization, an ERS is expected following an increase in cortical activation. Converesely, when the cortical activation baseline is high and the majority of neurons is occupied by synchronization processes, an increase of cortical activation can induce an ERD. The number of neurons available for synchronization and the excitation level of cortical neurons are the two parameters that define the WP in the cortical activation model and therewith the amplitude of oscillations. However, this still remains quite a theoretical concept if not included in a quantitative framewok, which can explain the relationship between the WP and the mechanism that generates a specific rhythm. Grabska-Barwinska proposed a simplified lumped computational model of a cortical circuit consisting only of pyramidal and fast spiking interneuronal populations. The model elucidates the mechanisms of transition between slower and faster rhythms, gamma synchronization and beta desynchronization and rebound effects during motor preparation and execution (Grabska-Barwińska and Żygierewicz, 2006).

The introduced works have analyzed different aspects of the same phenomenon, but they also exhibit important limitations, especially for what concerns the analysis in the beta band. In particular, a neural model which includes four populations is more adequate to analyze how beta rhythms are generated in the cortex (Wendling et al., 2002). Moreover, previous models do not include the interhemispheric communication and so cannot analyze its role in the ERD/ERS generation.

In order to overcome the previous limitations, in the present paper we make use of a NMM that: (i) generates beta rhythm through a four population modeling of intra-cortical networks. In particular, we used the four population NMM by Wendling et al. (2002), originally proposed to simulate rhythms generation in the hippocampus, but with parameters assigned to simulate beta rhythms in the right and left SMA (Zavaglia et al., 2006). (ii) evaluates how intercortical connections transmit that rhythm. Hence, the overall model consists of two neural columns (one per each area). For both the two areas, we defined a set of parameters to generate beta rhythm independently of one another. The intercortical connections are modeled as long range synapses that mimic the transcallosal connections (TC).

The final aim is to gain a deeper understanding of the mechanisms responsible for ERD/ERS in the somatosensory cortex in response to a modulating input. In particular, we aim (i) to verify whether the experimentally observed ERD/ERS patterns can be ascribed to a simple change of the population WP and to the TC transmission between the two hemispheres, (ii) to assess whether the obtained results support the well-known theory of inhibition. A more general aim is to provide a straightforward theoretical framework, which can be used to interpret different data (depending on the WP), to drive BCI experiments, and to formulate new testable predictions.

## Methods

### Single column model

**Figure 1:**
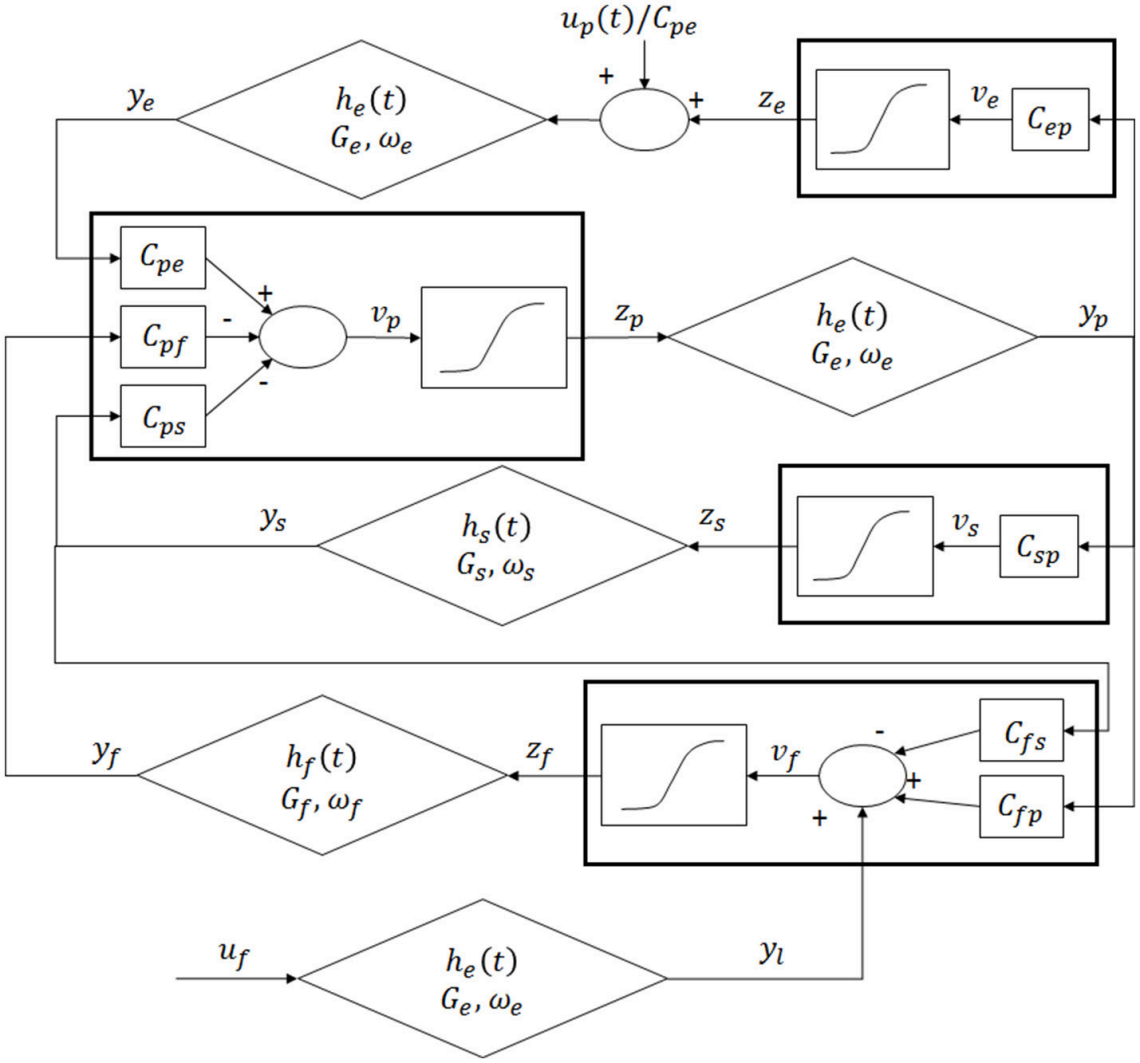
Layout of the model of a single region: four neural populations (pyramidal cells, excitatory interneurons, fast inhibitory interneurons and slow inhibitory interneurons) which communicate via excitatory and inhibitory synapses.

The model of a single area was obtained by modifying the Equations by Wendling et al. (2002). The model consists of four populations of neurons, pyramidal cells (p), excitatory interneurons (e), inhibitory interneurons with slow synaptic kinetics (s) and inhibitory interneurons with fast synaptic kinetics (f). Neurons in each population are lumped together and are assumed to share the same membrane potential. One lumped circuit communicates with another through the average firing rate, which corresponds to the average activity of that given population of cells. Each neural group receives an average postsynaptic membrane potential from the other groups, and converts the average membrane potential into an average density of spike fired by the neurons. This conversion is simulated via a static sigmoidal relationship. The effects of synapses are described by a second order linear transfer function, which converts the presynaptic spike density into the postsynaptic membrane potential. Three different kind of synapses, with impulse response *h*_*e*_, *h*_*s*_ and *h*_*f*_, are used to describe the synaptic effect of excitatory neurons (both pyramidal cells and excitatory interneurons), of slow inhibitory interneurons and fast inhibitory interneurons. The layout of the model of a single column is shown in Figure 1.

According to Figure 1, model Equations can be written as follows:

**PYRAMIDAL NEURONS**

(2.1) $$\frac{dy_{p}(t)}{dt}=x_{p}\;(t)$$

(2.2) $$\frac{dx_{p}(t)}{dt}=G_{e}\;\omega_{e}z_{p}\left(t\right)-2\omega_{e}x_{p}\left(t\right)-\omega_{e}^{2}y_{p}(t)$$

(2.3) $$z_{p}=\frac{2\;e_{0}}{1+\;e^{r(s_{0}-v_{p})}}$$

(2.4) $$v_{p}=C_{pe}y_{e}\left(t\right)-\;C_{ps}y_{s}\left(t\right)-\;C_{pf}y_{f}\left(t\right)$$

**EXCITATORY INTERNEURONS**

(2.5) $$\frac{dy_{e}(t)}{dt}=\;x_{e}\;(t)$$

(2.6) $$\frac{dx_{e}(t)}{dt}=G_{e}\;\omega_{e}\left(z_{e}\left(t\right)\text{​}+\text{​}\frac{u_{p}\left(t\right)}{C_{pe}}\right)\text{​}-\text{​}2\omega_{e}x_{e}\left(t\right)\text{​}-\text{​}\omega_{e}^{2}y_{e}(t)$$

(2.7) $$z_{e}=\frac{2\;e_{0}}{1+\;e^{r(s_{0}-v_{e})}}$$

(2.8) $$v_{e}=C_{ep}y_{p}\left(t\right)$$

**SLOW INIBHITORY INTERNEURONS**

(2.9) $$\frac{dy_{s}(t)}{dt}=\;x_{s}\;(t)$$

(2.10) $$\frac{dx_{s}(t)}{dt}=G_{s}\;\omega_{s}z_{s}\left(t\right)-2\omega_{s}x_{s}\left(t\right)-\omega_{s}^{2}y_{s}(t)$$

(2.11) $$z_{s}=\frac{2\;e_{0}}{1+\;e^{r(s_{0}-v_{s})}}$$

(2.12) $$v_{s}=C_{sp}y_{p}\left(t\right)$$

**FAST INIBHITORY INTERNEURONS**

(2.13) $$\frac{dy_{f}(t)}{dt}=\;x_{f}\;(t)$$

(2.14) $$\frac{dx_{f}(t)}{dt}=G_{f}\;\omega_{f}z_{f}\left(t\right)-2\omega_{f}x_{f}\left(t\right)-\omega_{f}^{2}y_{f}(t)$$

(2.15) $$\frac{dy_{l}\left(t\right)}{dt}=\;x_{l}\;\left(t\right)$$

(2.16) $$\frac{dx_{l}(t)}{dt}=G_{e}\;\omega_{e}u_{f}\left(t\right)-2\omega_{e}x_{l}\left(t\right)-\omega_{e}^{2}y_{l}(t)$$

(2.17) $$z_{f}=\frac{2\;e_{0}}{1+\;e^{r(s_{0}-v_{f})}}$$

(2.18) $$v_{f}=C_{fp}y_{p}\left(t\right)-\;C_{fs}y_{s}\left(t\right)+\;y_{l}$$

**Figure 2:**
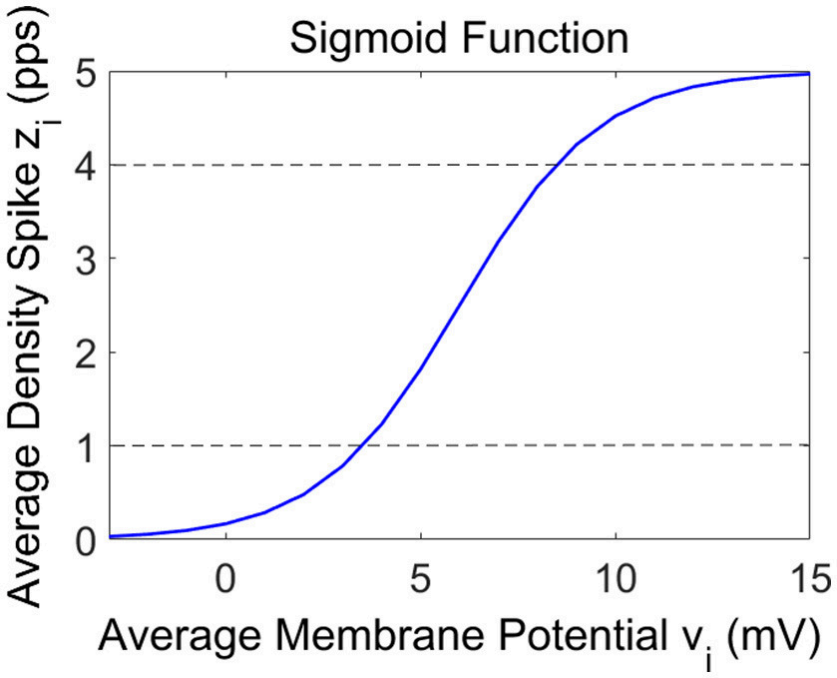
Representation of the sigmoid curve used in the model.

In these Equations and in Figure 1, the symbols *v*_*i*_ represent the average membrane potentials (*i* = *p, e, s, f*). These are the inputs for the sigmoid function which converts them into the average density spike (*z*_*i*_, *i* = *p, e, s, f*) fired by the neurons. The sigmoid function is defined by parameters *e*_0_, *s*_0_ and *r*, and it is shown in Figure 2. These parameters, assumed to be equal for all populations, set the maximal saturation, the position, and the slope of the sigmoid, respectively. In the curve, we identified a low saturation region (*z*_*i*_ <1), a linear slope region (1 < *z*_*i*_ < 4) and a high saturation region (*z*_*i*_ > 4).

These outputs (*z*_*i*_) enter into the synapses (excitatory, slow inhibitory or fast inhibitory), represented by the second order linear functions. Each synapse is described by an average gain (*G*_*e*_, *G*_*s*_, *G*_*f*_ for the excitatory, slow and fast inhibitory synapses, respectively) and a time constant (the reciprocal of ω_*e*_, ω_*s*_ and ω_*f*_, respectively). The outputs of these Equations represent the postsynaptic membrane potentials (*y*_*i*_, *i* = *p, e, s, f*). Interactions among neurons are represented via seven connectivity constants *C*_*ij*_, from the j-th population to the i-th one. Finally *u*_*i*_ (*i* = *p, f*) represents the external input to the column, which will be described in detail in paragraph Driving Input.

Since the beta frequency band in which ERD/ERS occur may differ among individual subjects, we tested the robustness of the obtained results by using three sets of parameters, to simulate three beta sub-bands. Specifically, starting from the parameters' values proposed by Zavaglia et al. (2006) we finely tuned synapsis average gains and time constants to obtain three power spectra with the peaks in. low beta (LB: 14–19 Hz), medium beta (MB: 20–24 Hz) and high beta (HB: 25–30 Hz) sub-bands. The values of these parameters are reported in Table 1.

**Table 1:** Model basal parameters.

**Common parameter**	**Low beta**	**Medium beta**	**High beta**
*C* = 135			
*C*_*ep*_ = *C*	*G*_*e*_ = 3.9	*G*_*e*_ = 3.9	*G*_*e*_ = 4.3
*C*_*pe*_ = 0.8 *C*	*G*_*s*_ = 4.3	*G*_*s*_ = 4.3	*G*_*s*_ = 4.6
*C*_*sp*_ = 0.25 *C*	*G*_*f*_ = 25	*G*_*f*_ = 25	*G*_*f*_ = 29
*C*_*ps*_ = 0.25 *C*	ω_*e*_ = 55	ω_*e*_ = 75	ω_*e*_ = 90
*C*_*fp*_ = 0.3 *C*	ω_*s*_ = 25	ω_*s*_ = 33	ω_*s*_ = 36
*C*_*fs*_ = 0.1 *C*	ω_*f*_ = 250	ω_*f*_ = 330	ω_*f*_ = 380
*C*_*pf*_ = 0.8 *C*			
*s*_0_ = 6			
*e*_0_ = 2.5			
*r = 0.56*			

### Two columns model and transcallosal connection

#### Transcallosal connection

**Figure 3:**
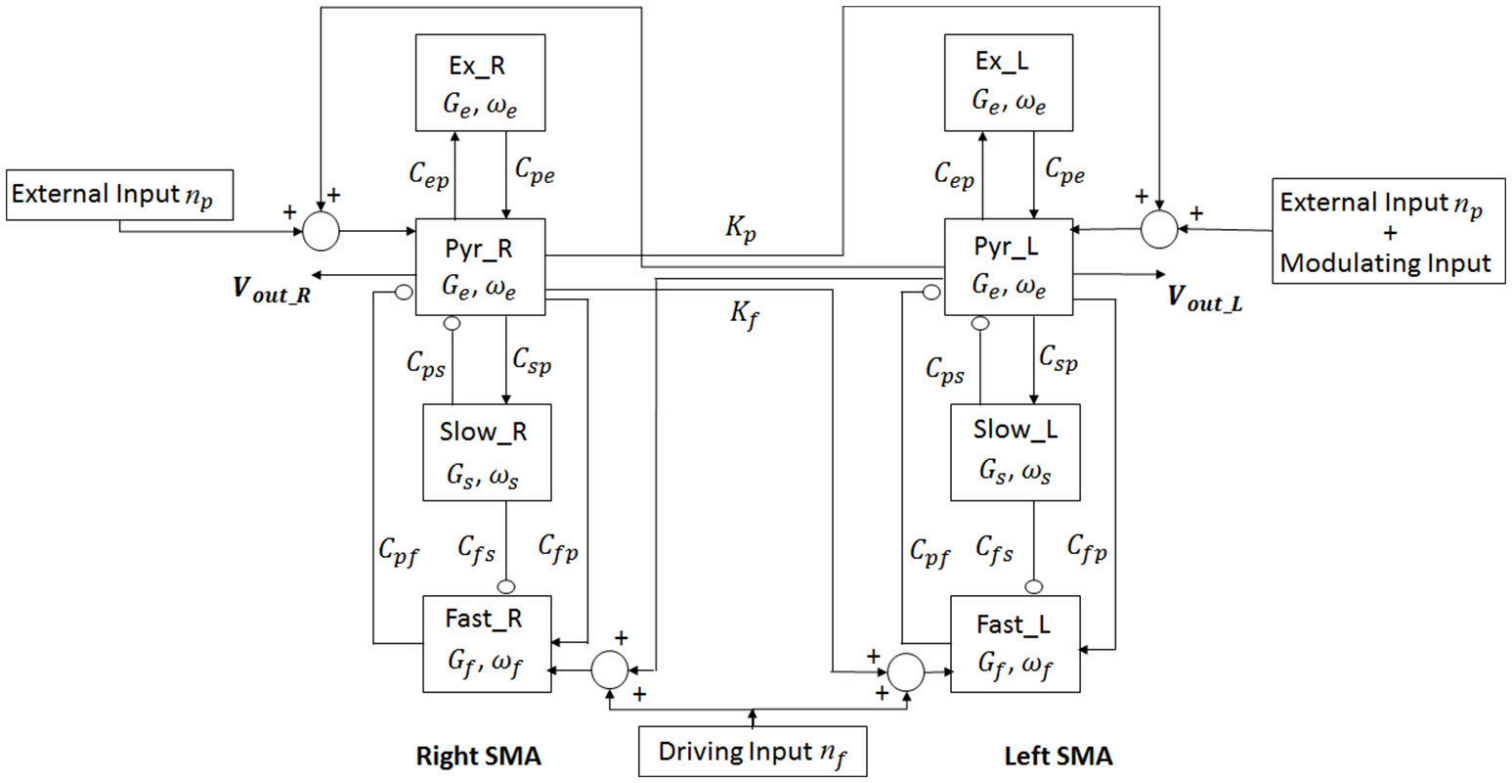
Layout of the model of two regions. For each region (Right and Left SMA), four neural populations (pyramidal cells, excitatory interneurons, slow-inhibitory interneurons and fast-inhibitory interneurons) communicate via excitatory and inhibitory synapses. The connection between the two cortexes is mediated by two connectivity constants which origin from pyramidal neurons of one cortex and target the pyramidal neurons and the fast-inhibitory interneurons of the other cortex.

In order to study the interhemispheric connectivity by TC, we considered two cortical areas, the right and the left SMA (each described by Equations 2.1–2.18), interconnected through long-range excitatory connections with a time delay. Figure 3 shows the model layout.

In TC modeling, we relied on the assumptions that all transcallosal fibers are excitatory at a neurochemical level (Bloom and Hynd, 2005) since the long-range connections originate exclusively from pyramidal population. However, both excitatory and inhibitory messages have been demonstrated to travel through the corpus callosum (Swayze, 1987; Bloom and Hynd, 2005); indeed, the functional effects of these connections depend on various factors, such as the receptors and the interneurons involved. In particular, a synaptic connection from pyramidal neurons in one area to pyramidal neurons in the other areas has an excitatory role on the target region, whereas a connection from pyramidal neurons in one area to inhibitory interneurons in the other area has an inhibitory effect.

Ursino et al. demonstrated that an excitatory connection to fast inhibitory interneurons is able to transmit the rhythms from one region to the other very efficaciously, while connections to slow inhibitory interneurons are less effective (Ursino et al., 2010). For these reasons, we modeled TC as projections from pyramidal cells to contralateral pyramidal neurons and to fast inhibitory interneurons.

To simulate connectivity, we assumed that the average spike density of pyramidal neurons of the presynaptic area (*z*_*p*_) affects the target region via a connectivity constant to fast inhibitory interneurons (*K*_*f*_) and to pyramidal neurons (*K*_*p*_) with a time delay *T*.

This is achieved by modifying the input quantities *u*_*p*_ and *u*_*f*_ of the two cortexes.

We can write:

(2.19) $$u_{i}(t)=n_{i}(t)+K_{i}\;\cdot \;z_{p}(t-T)i=p,\;f$$

where *n*_*i*_(*t*) represents the external input (see Section Driving Input).

In the model, we defined a global connectivity constant *K* as:

(2.20) $$K=K_{p}+\;K_{f}$$

We used the same value of *K*_*p*_ and *K*_*f*_ for both cortexes. To assign a relative value for these parameters, we made two approximations: (i) we considered only layers 2/3 and 5, provided that the origins of TC is mainly located in those layers (Koralek et al., 1990; Rouiller et al., 1991; Martínez-García et al., 1994; Karayannis et al., 2007; Molyneaux et al., 2007); (ii) we considered that the fibers from layers 2/3 and 5 generate more synapses to fast-spiking interneurons (about 70%) than to pyramidal neurons (about 30%). The contribution of synapses to the other interneurons was considered negligible (Dantzker and Callaway, 2000).

Accordingly, we assumed that 70% of synapses target the fast inhibitory interneurons (hence *K*_*f*_ = 0.7*K*), while the remaining 30% of synapses target the pyramidal ones (*K*_*p*_ = 0.3*K*). The choice of these percentages might seem decisive for model behavior. However, a preliminary sensitivity analysis showed that the overall behavior of the model does not change appreciably if this ration is changed even greatly. Indeed, only the edges of the working regions range, that will be described below, slightly changes.

It is known that the corpus callosum has a variable number of fibers and, depending on the considered theory (Theory of inhibition or Theory of Excitation), that number correlates negatively/positively with the information transfer and positively/negatively with the lateralization, respectively. To represent this variability, we tested the model with 101 *K K*-values equally distributed between 0 and 100; then *K*_*p*_ varies between 0 and 30 and *K*_*f*_ between 0 and 70.

The delay in the information transfer between the two cortexes, which depends on the complexity of the specific task, can vary in a range 10–300 ms. Considering the results of transcranial magnetic stimulation studies, transcallosal conduction times between motor cortexes of healthy subjects are typically about 13 ms (Liederman, 1998). So we used a time delay *T* equal as great as 13 ms.

#### Driving input

Parameters sensitivity analysis showed that inputs to slow inhibitory and excitatory interneurons do not produce appreciable changes in the model dynamics (Ursino et al., 2010). Therefore, in the following, we will consider only external inputs to pyramidal neurons and to fast inhibitory interneurons.

The external input *n*_*i*_(*t*) (*i* = *p, f*) is represented as a Gaussian white noise which accounts for all inputs not incorporated in the model, both excitation coming from the environment and the density of action potentials coming from other connected regions. It was modeled as a positive input to the pyramidal cells (hence excitatory) with mean *m* = 40 pps (pulses per second) and standard deviation σ = 1 and a positive input to the fast inhibitory interneurons (hence with an inhibitory effect) with *m* = 3 pps and σ = 1. The external inputs have the same *m* and σ for the two cortexes. The inputs are given for the entire simulation. The model parameters and the external input set the WP at a level similar to that used by other models in literature (Wendling et al., 2002; Zavaglia et al., 2006).

The driving input has a very important role for setting the population WP and then the potential cortex excitability. In particular, in agreement with the cortical activation model (Pfurtscheller, 2006), the baseline WP determines the modulation of the cortex, and so the induction of an ERD or an ERS.

#### Modulating input

To simulate the occurrence of the task, consisting in the persistent imagery of the right hand movement, we added a modulating input to the pyramidal population of the contralateral cortex (i.e., to the left SMA).

**Figure 4:**
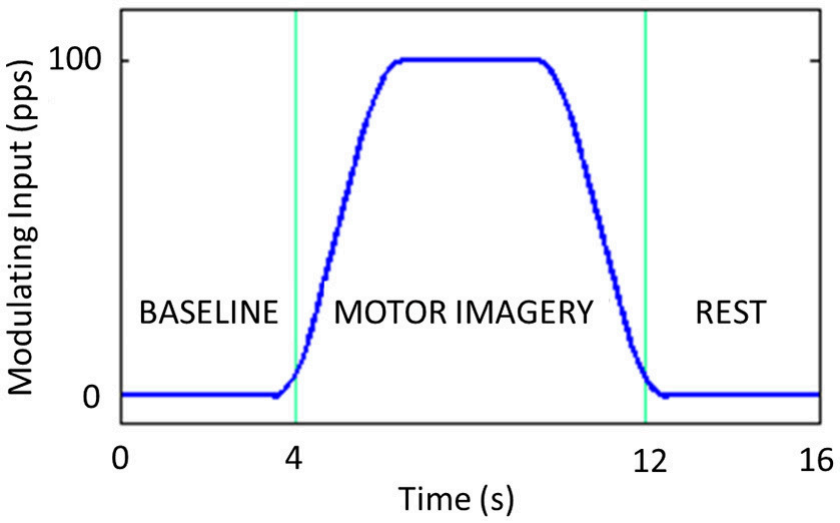
Modulating input introduced to the Left SMA. It includes 4 s of baseline, 8 s of motor imagery and 4 s of rest.

We simulated a typical motor imagery trial lasting 16 s: 4 s of baseline, 8 s of motor imagery and 4 s of rest (Suffczynski et al., 2001). The modulating input is modeled as a smoothed trapezoidal function, with the rise and fall times as long as 2 s and a maximum amplitude plateau as high as 100 pps lasting 4 s (Figure 4).

#### Model output

Outputs of the model are the average membrane potentials of the pyramidal populations, which simulate the local field potential recorded by superficial EEG over the left and the right SMA (in Figure 3 *v*_*out*_*L*_ and *v*_*out*_*R*_, respectively). Moreover, during the simulations we also analyzed the average spike density *z*_*p*_ of the pyramidal neurons. This variable is related with the neuronal firing rate, then it contains a direct information on the interaction between the two cortexes. In particular, *z*_*p*_ can vary between 0 and 2 × *e*_0_. In the following, we will call WP the value of *z*_*p*_ around which the cortex works. The simulated sample frequency is 100 Hz.

After introducing the modulating input and once fixed the WP, we computed the ERD/ERS as follows:

(2.21) $$ERD/ERS\left(\%\right)=\frac{P\left(t\right)-P_{B}}{P_{B}}\;\cdot \;100$$

where *P*(*t*) is the power extracted of *v*_*p*_(*t*) at each time-point over the 16 s, and *P*_*B*_ the mean power in baseline (during the first 4 s).

## Results

### Single column model

**Figure 5:**
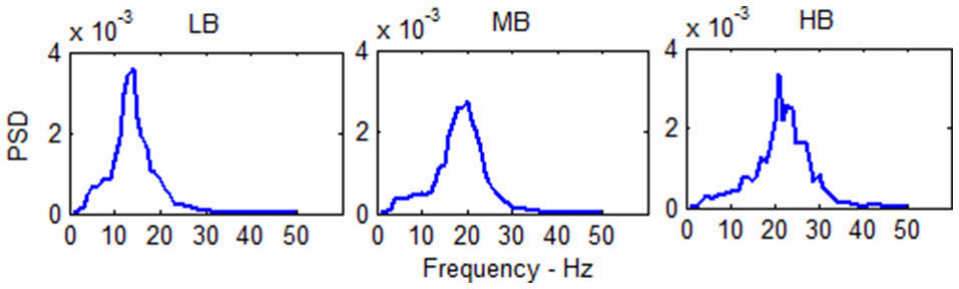
PSD of the single column model output in the three beta sub-bands (Low Beta, Medium Beta and High Beta). The PSD spectra are computed with modified periodogram method using 1 s window and 0.01 s overlapping and averaged for the total length of the trial.

The power spectral density (PSD) in the three beta sub-bands is shown in Figure 5.

The spectra show a peak at three different frequencies (about 15, 20, and 25 Hz) covering the all range of beta frequencies. As reported in Table 1, it is possible to modulate the spectral content and obtain the generation of these rhythms just tuning *G*_*i*_ and ω_*i*_, *i* = *e, s, f*.

The three spectra are the starting point to evaluate the changes induced by the connection of the two cortexes. As in the previous presented models (Wendling et al., 2002; Zavaglia et al., 2006), the WP of the pyramidal population for the three beta sub-bands is around 4, then near to the transition between the linear slope region and the high saturation region (Figure 1). The excitatory interneurons population works in a condition of completely upper saturation (WP = 5), the slow inhibitory interneurons work near the linear region and the upper saturation region (WP = 3/4) and the fast inhibitory interneurons work near the low saturation region (WP = 0.5/0.8). These working conditions make the fast inhibitory interneurons the most sensitive population to an excitatory driving input because, starting from a lower saturation condition, they have the greater margin to increase and to move their WP near a linear region.

### Two columns model and transcallosal connection

#### Driving input

For each beta sub-bands, we considered 101 values of *K* to evaluate the role of the connection strength on the transmission of the signal from one cortex to the other. In this phase there is no modulating input on the cortexes.

**Figure 6:**
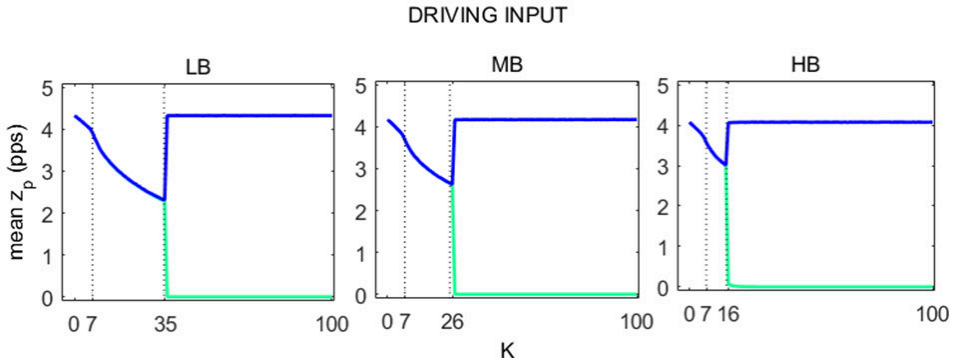
Trends of pyramidal average spike density for both Right (blue line) and Left (green line) SMA as a function of *K*. For each of the three beta sub-bands three regions were identified, here delimited with a dashed line.

The average value of *z*_*p*_ over time (16 s), for each of the two cortexes, is shown in Figure 6 as a function of *K*. LB, MB, and HB curves show the same behavior and include 3 regions:

- In the first region the relation between mean *z*_*p*_ and *K* describes a concave curve, which reflects the passage from the saturated to the linear slope region of the sigmoid.
- In the second region, the relation between mean *z*_*p*_ and *K* describes a convex curve. As for the first region, the average *z*_*p*_ decreases with *K*, moving in the linear slope region of the sigmoid function.
- In the third region, the activity in the two cortexes quickly move away from the linear slope region of the sigmoid. In particular, activity in one region falls to zero, i.e., the WP moves in the silent region of the sigmoid, while the other cortex works in its original WP, as if there were no connection with the other cortex.

The previous results can be explained by the following mechanisms: in the first two regions, the network start from an upper saturation point and the presence of TC move the WP of pyramidal neurons near a linear region of the sigmoid, increasing the interhemispheric communication. In particular, excitation from pyramidal neurons of the other hemisphere, via the inter-hemispheric connection *K*_*f*_, causes excitation of fast inhibitory interneurons. Indeed, this population has a very important role since it is sensible to the input and produces the greater variations of the WP at the increase of *K*. Conversely, excitation to pyramidal neurons, through the connection *K*_*p*_, has a less relevant role, since this population works close to the upper saturation. The strong increase in *z*_*f*_, in turn, causes a significant inhibition of pyramidal neurons, with the global result to move the pyramidal WP toward a linear region of the sigmoid. Moreover, the decrease of pyramidal activity reduces the WPs of the slow inhibitory interneurons and of excitatory interneurons. Finally, it is worth while that a reduction in the WP (i.e., functioning in the linear region) is associated with larger fluctuations in the population activity induced by noise.

The behavior in the third zone reflects a competitive mechanism, similar to a winner takes all dynamics: only one region (due to a better influence of external noise) wins the competition causing the almost complete inhibition of the other. This mechanism reflects a strong lateralization of the brain.

**Figure 7:**
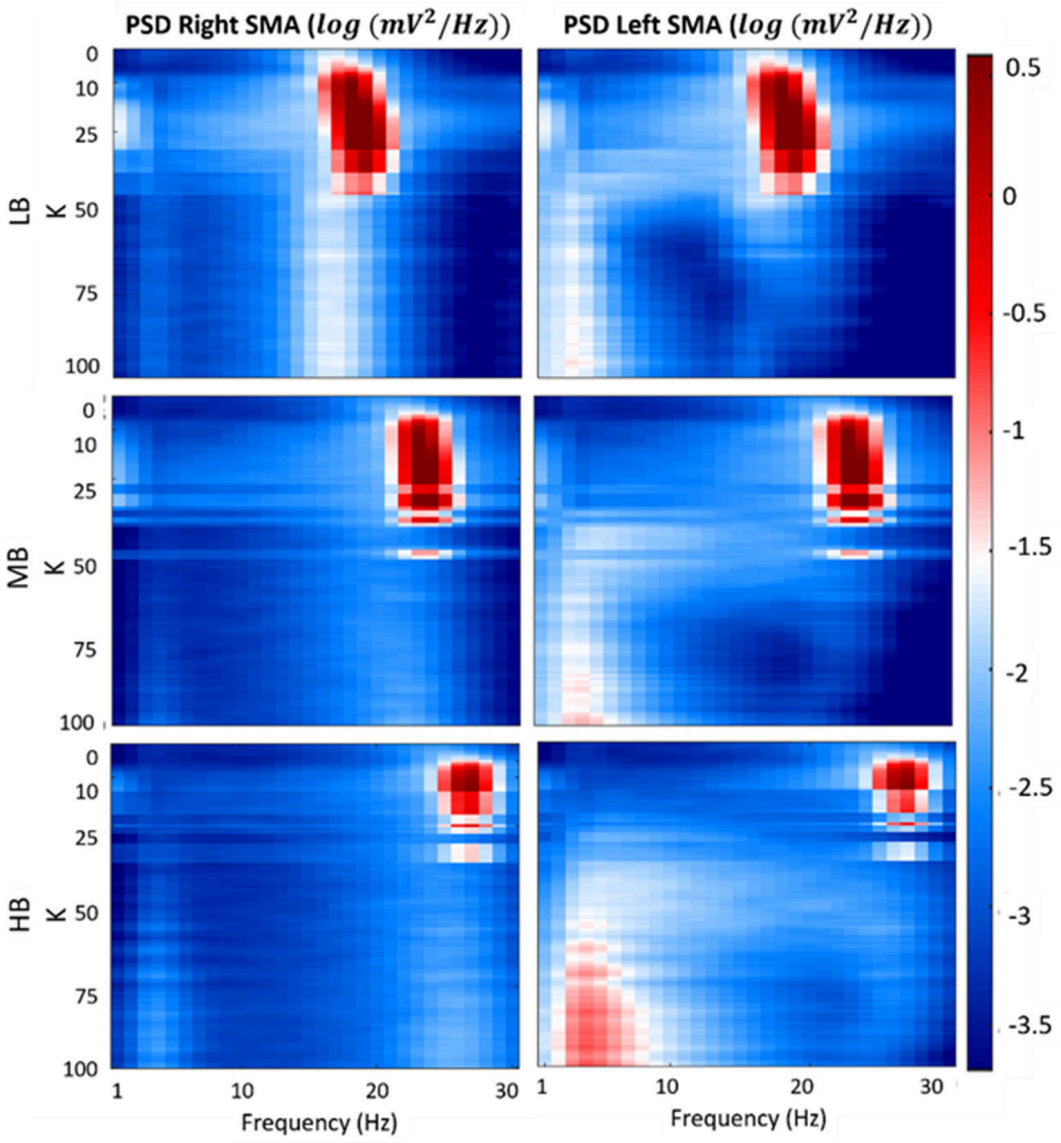
Plots of the log-transformed PSD extracted from both cortexes for each beta sub-band and for each *K*-value. The PSD spectra are computed with modified periodogram method, using 1 s window and 0.01 s overlapping and averaged for the total length of the trial.

Figure 7 shows a map of the *v*_*out*_*R*_ and *v*_*out*_*L*_ log-transformed PSD spectra of each beta sub-bands, computed for each connectivity constant *K*. In the figure, the three previously identified regions are well evident for each beta band. Specifically, when *K* is lower than 7, the maps show the main spectral content in the beta bands (light blue areas) for both the cortexes. Similar to the first region, values of *K* belonging to the second identified region (LB: 7 < *K* < 35, MB: 7 < *K* < 26 and HB: 7 < *K* < 16), provide PSD spectra with the main content in the beta bands but with a greater amplitude (red areas) for both right and left SMA. In the third region (LB: *K* > 35, MB: *K* > 26 and HB: *K* > 16), one of the cortex is completely inhibited (left SMA) and it shows the main spectral content in low frequency band (2–7 Hz) (light red and white areas), whereas the other cortex shows a behavior as in the “no-connectivity” condition with the main spectral content in beta bands (light blue and white areas).

**Figure 8:**
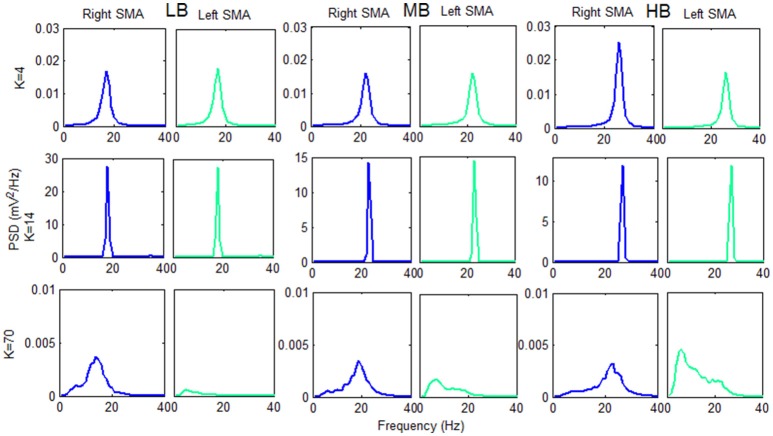
Plots of the PSD extracted from both cortexes for each beta sub-band using three *K*-values belonging to the three previously identified working regions. The PSD spectra are computed with modified periodogram method, using 1 s window and 0.01 s overlapping and averaged for the total length of the trial.

To clarify this phenomenon Figure 8 shows the *v*_*out*_*R*_ and *v*_*out*_*L*_ PSD spectra of each beta sub-bands, computed using 3 connectivity constants belonging to the 3 above identified working regions.

The information transfer is well evident using the connectivity constant *K* = 4: the PSD spectra of the two cortexes are similar and have a power content greater that the “no-connectivity” condition (Figure 5).

Using *K* = 14 there is a very efficient transmission of the two rhythms: the two cortexes are indeed synchronized at the same frequency, and the PSD spectra show a peak of higher amplitude with respect to the *K* = 4 condition.

In the third region (*K* = 70), we found a different behavior between the two cortexes: one cortex works as in the “no-connectivity” condition, whereas the other is characterized by a low frequency content. This signifies that the strong connection strength form the first to the second cortex causes a slower rhythm (mainly in the theta range) but, due to the prevalence of inhibition, the average membrane potential in the second cortex lies below the threshold of the sigmoid relationship, i.e., the second region works in the low saturation region.

#### Modulating input

After analyzing the behavior of the system with the driving input alone, we added the modulating input to simulate the imagery task. Since simulation concerns the imagination of the right hand movement, we gave the input to the left SMA.

**Figure 9:**
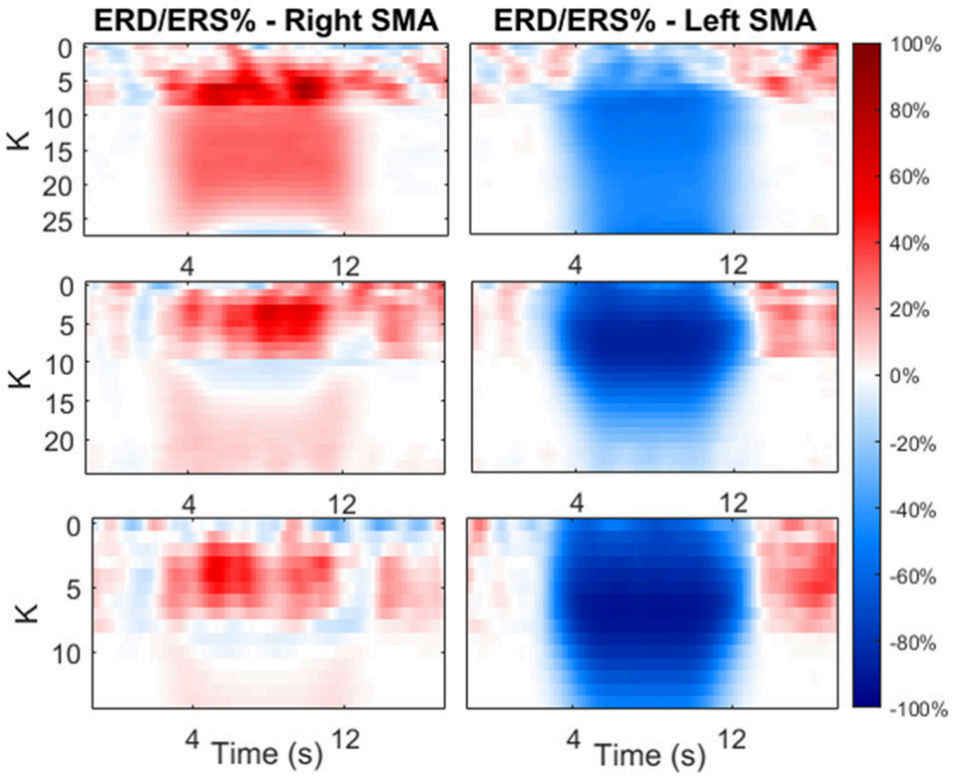
Maps of ERD/ERS % as function of time during the modulating input and as function of *K*-value. The value of *K* was varied only in the first two previously identified regions. The maps are reported for both cortexes and each beta sub-band.

Figure 9 shows the ERD/ERS % maps (computed with Equation 2.21) as a function of the time and as function of *K*. In this phase, we focused on the first two previously identified *K* regions.

As shown in the figure, an increase in *K* causes a significant decrease in the average power (hence ERD) in the contralateral side, and a significant increase in power (hence ERS) in the ipsilateral side. The latter increases with *K* until a maximum is reached, than the effect decreases at larger values of *K*.

**Figure 10:**
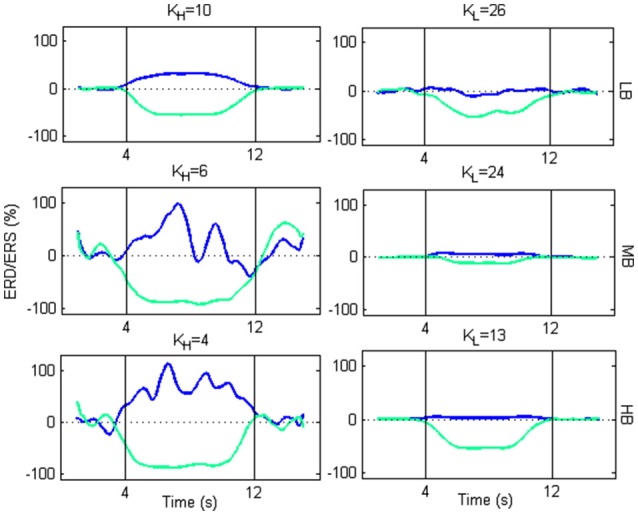
Plots of ERD/ERS % as function of the time during a trial. For each beta sub-band, the selected *K*_*H*_ and *K*_*L*_ are used to extract the ERD/ERS.

From Figure 9, we selected two *K K*-values, *K*_*H*_ and *K*_*L*_, for each beta sub-bands, corresponding to a high and low amplitude of ERD/ERS, respectively, and tested the temporal pattern of ERD/ERS during the task (Figure 10). The selected values are 10 and 26 for LB band, 6 and 24 for MB band and 4 and 13 for HB band.

For each beta sub-bands the figure shows that, using *K*_*H*_, an ERD occurs in the Left SMA, while an ERS occurs in the Right SMA, at the beginning of the imagery task. As the motor imagery task ends, the two powers return to their baseline values. Using *K*_*L*_, the transmission becomes less efficient. Actually, the ERD follows the same behavior as for *K*_*H*_, but with a lower amplitude. Conversely, the ERS is not generated.

**Figure 11:**
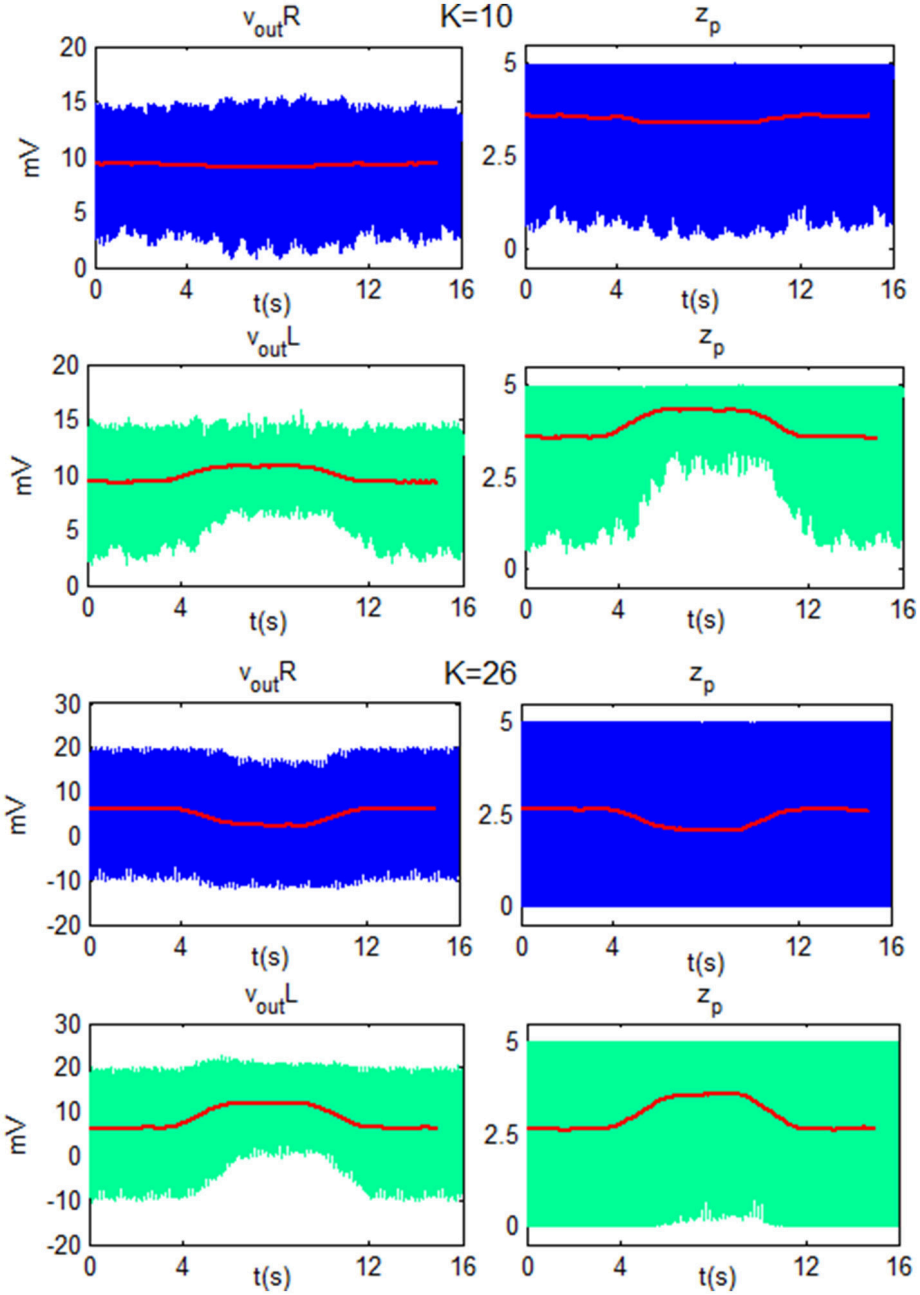
Plots of *v*_*out*_ and *z*_*p*_ of the Right cortex (blue line) and of the Left SMA (green line) as function of time for LB using *K* = 10 and *K* = 26. For both *K*-values, the modulating input moves *z*_*p*_ of the target cortex (left) toward the upper saturation region and so the amplitude of oscillation decreases. For *K* = 10, *z*_*p*_ in the ipsilateral cortex (right) moves toward a linear region and the amplitude of the oscillation increases. For *K* = 26, *z*_*p*_ in the ipsilateral cortex has already reached a maximum oscillation amplitude, then it cannot be further modulated and *v*_*out*_*R*_ does not change its amplitude. The red line is the moving average filtered signal of each subplot.

To better explain the ERD/ERS generation, some exempla of *v*_*out*_*R*_, *v*_*out*_*L*_, *z*_*p*_*R*_ and *z*_*p*_*L*_ are showed as function of time in Figure 11. The figure shows the behavior of the network for low beta and the two *K*-values used in Figure 9 (*K*_*H*_ = 10 and *K*_*L*_ = 26). In particular, the ERD is generated for both *K*-values since the modulating input, during the motor imagery task, moves *z*_*p*_ of the target cortex (left cortex) toward upper saturation. The smaller fluctuations in *z*_*p*_, in turn, are reflected in smaller fluctuations in the overall column, and so the amplitude of *v*_*out*_*L*_ decreases. For *K*_*H*_, the TC produces a decrease of *z*_*p*_ in the ipsilateral cortex, (right), moving the WP in the linear region. This corresponds to large fluctuations (ERS). For *K*_*L*_, *z*_*p*_ oscillation of the right cortex is already maximum and it cannot generate a further ERS.

## Discussion

### Two columns model and transcallosal connection

We used a neural mass model to simulate the connection between the Left and the Right SMA in resting state and during a motor imagery task. In particular, we adopted the model by Wendling et al. (2002) which represents a good compromise between synthesis and completeness. Compared with the well-known model by Jansen and Rit (1995) it incorporates a fourth population of fast inhibitory interneurons, which allows a more accurate simulation of brain rhythms especially at high frequencies (Beta and Gamma bands). We did not include the thalamus, as in other recent models (Suffczynski et al., 2001; Cona et al., 2014), since a thalamic feedback is especially significant in the generation of lower frequency rhythms (Alpha and Theta) which are not investigated in the present work.

The communication between the two cortexes is driven by means of TC, simulated as a connectivity constant, which transmits the cortical rhythms (Pyramidal Output), using the same dynamics used for the excitatory (glutamatercig) synapses. This choice agrees with the well-accepted idea that long-range connections are imputable to pyramidal neurons. Furthermore, we included a physiological time delay to account for the long inter-hemispheric connection.

A difficult problem is to decide to which population the connection should target. Neurophysiological data (Felleman and Van Essen, 1991) suggest that long range connections from pyramidal neurons to distal cortical regions can reach all populations, depending on the role of these connections. However, a previous work using a similar NMM (Ursino et al., 2010) showed that synapses to excitatory interneurons and to slow inhibitory interneurons have a minor influence on the dynamics of the target region. Hence, for the sake of parsimony, only pyramidal-pyramidal and pyramidal-fast inhibitory connections are included in this work. Furthermore, according to neurophysiological data, we assumed that connections to inhibitory neurons are more numerous than connections to the pyramidal ones. We explored a 0–100 range of connectivity constants *K*, which simulate the TC strength.

#### Driving input

Analyzing Figure 6, we distinguished 3 regions:

- In the first region the neurons work close to the upper saturation, and so there is only a moderate communication of the individual rhythms.
- In the second region, the two neurons work in the linear slope region of the sigmoid function, since the reciprocal inhibition reduces the position of the WP. This is the region of maximum information transfer because the two cortexes' WPs are near the sigmoid central point, and so the oscillatory activity is maximal.
- In the third region, one of the cortexes works below the lower threshold of the sigmoidal relationship (mean *z*_*p*_ close to 0) while the other works at its own equilibrium point (around *z*_*p*_ = 4). In this condition, one cortex is completely inhibited, while the other is potentially excitable and its WP is the same as for *K* = 0.

Behavior in the third region agrees with the inhibitory theory of metacontrol proposed by Banich. In fact, in metacontrol information presented to both hemispheres is completely managed by one dominant hemisphere (Banich, 1995). In other words, by inhibiting activity of the opposing hemisphere the other hemisphere becomes dominant for the processing of the stimulus information (Hellige et al., 1989). The model suggests a similar behavior in case of high values of *K* (i.e., in the third region described above). Here, model behavior implements a “winner takes all” dynamics, in which only one among the competitors (in this case one of the two areas of the cortex) wins the competition and completely dominates the task. Conversely, at intermediate vales of K, both hemispheres are involved in the process, and in this region we can observe the maximal synchronization between the two rhythms. Here, the model suggests that cooperation between the two hemispheres is the adopted strategy, in which binding of the rhythms allows the integration of the two pieces of information (left and right) into a single combined response.

#### Modulating input

In this section we will discuss how a modulating input is transmitted from the target cortex to the other. When *K* is equal to zero (no connection) the input is not transferred from one cortex to the other, than an ERD is generated on the target cortex but and ERS is not generated in the controlateral one. However, for low values of *K* the transfer is maximum (blue and red areas for ERD and ERS, respectively in Figure 8) and specifically there is a turning point after which, increasing the value of K, the transfer starts to decrease (light blue and light red areas for ERD and ERS, respectively in Figure 8). The condition of maximum transfer is a condition in which the two cortexes are near the transition from the concave region to the convex region. In this point the *z*_*p*_ amplitude and mean are not too high, so a modulating input is able to modulate the *z*_*p*_ in a significant way.

Our simulation results show that a modulating input produces a decrease of the power in beta band on the target cortex ERD and an increase of the power on the ipsilateral cortex ERS. This can be explained with the following mechanism. The input excites the target cortex, which inhibits more the ipsilateral cortex. The latter in turn inhibits less the target cortex. This process generates a loop to which the target cortex is increasingly excited and the ipsilateral one increasingly inhibited. As a consequence, the WP of the target cortex moves to a position closer to the upper saturation, i.e., a position where the amplitude of the rhythm decreases. In fact, here the spiking frequency of the neurons is less affected by the external noise. Conversely, the WP of the ipsilateral cortex moves down in the linear region of the sigmoid relationship, where the spiking frequency of neurons is maximally affected by noise, thus causing a power increase in the beta range. This mechanism allows the generation of the ERD (related with an excitation state, close to upper saturation) and ERS (related with a inhibition state, close to the central region) patterns.

Of course, this result strongly depends on model assumption on the initial WP of the two pyramidal populations, placed in the upper portion of the sigmoidal relationship, not too distant from saturation (Wendling et al., 2002; Zavaglia et al., 2006) and is consistent with the model of cortical activation (Pfurtscheller, 2006). According to this theory, when the cortical activation baseline level is high and the majority of neurons is occupied by synchronization processes, an increase of cortical activation, induced for example by an external excitatory input, can only induce an ERD. This is the behavior observed in the *target cortex*. In turn, the WP of the pyramidal population strongly depends on WP of the fast interneurons population, placed in the lower saturation portion of the sigmoid. These neurons are very sensitive and ready to spike as soon as they receive an input. As a consequence, excitation of fast inhibitory neurons in the *ipsilateral cortex* (induced by TC from the target other cortex) causes a decrease in the WP of their pyramidal neurons, thus inducing an increase in fluctuations. This is in accordance with the cortical activation model: when the cortical activation baseline level is high and the majority of neurons is occupied by synchronization processes, a decrease of cortical activation can only induce an ERS.

In this phase we have not considered the before identified third region, since in that region one of the cortexes takes the control of the process and the other is completely turned off.

#### Excitation and inhibition theories

In the context of the debate about excitatory or inhibitory theories of the corpus callosum, this model is collocated mainly in accordance with the inhibitory theory.

If there are no modulating inputs on the cortex, but only a common driving input, as *K* increases, the interaction between the two cortexes moves the WP toward a more linear region of the sigmoid. After a certain value of *K*, i.e., increasing the strength of the TC, the laterality increases because one hemisphere is inhibited and the other tasked with the performance of the specific cognitive process. This is in accordance with the finding of Fling et al., which reported a strong, positive relationship between the strength of interhemispheric inhibition and the microstructure of interhemispheric fibers that is specific to tracts connecting the primary motor cortexes. In particular, an increased fiber microstructure in young adults predicts interhemispheric inhibitory capacity (Fling et al., 2013).

With the introduction of the modulating input, the behavior remains of inhibitory type: the inhibition of one cortex leads to the excitation of the other cortex, and vice versa. This behavior, causing an opposite shift in the WP of the two areas, can explain the observed ERD in the target cortex and the observed ERS in the ipsilateral cortex, and enforces the inhibitory theory: the two hemispheres are in constant mutually inhibitory relationship with each other and this competition is mediated by the corpus callosum. Also, included in this theory there is the notion that the corpus callosum serves as an “inhibitory barrier” between hemispheres to prevent maladaptive cross talk between the hemispheres for which a given function is dominant (Kinsbourne, 1982; Bloom and Hynd, 2005; Welcome and Chiarello, 2008; Adam and Güntürkün, 2009).

#### Implication in BCI control and new perspective

As previously investigated by several researchers (Filippini et al., 2010; Lindenberg et al., 2012; Halder et al., 2013), the integrity and the number of connections of the deep white matter structure are predictors of SMR-based BCI performance, and there is a relation between the white matter architecture and SMR-BCI aptitude. In particular, the corpus callosum was one of the top five white matter regions which were found to be most discriminating in the low vs. high BCI-aptitude group comparison, showing significant correlations (*p* < 0.05) with individual BCI-performance. According to our findings, when corpus callosum had a too low number of fibers, we expected a lower ERD and ERS amplitude and consequently lower BCI performances. At the increasing of corpus callosum fibers number, the generation of ERD/ERS becomes optimal until reaching a too high number of fiber and only an ERD is produced.

Halder et al. proposed that the best strategy to improve BCI performance in low aptitude users is by conducting a long-term BCI training program consisting of multiple sessions, which not only targets to increase proficiency in BCI usage for communication and control, but also attempts to incorporate interventions to increase or stabilize the microstructural integrity of BCI-critical central white matter (Halder et al., 2013).

In this context and in the light of our findings, we propose a further strategy to be combined with the one just presented. It consists in the use of neuromodulation techniques during BCI training (Matsumoto et al., 2010; Wei et al., 2013; He et al., 2014). In particular, an appropriate stimulation could move the neural circuit in an ideal WP, which could improve the information transfer between the two cortexes and increase the ERD/ERS amplitudes.

#### Model validation hypothesis

The validation of a model requires predictive ability to be tested. As the proposed model is based on several assumptions separately validated in the previously discussed studies (Pfurtscheller and Neuper, 1997; Suffczynski et al., 2001; Wendling et al., 2002; Grabska-Barwińska and Żygierewicz, 2006; Zavaglia et al., 2006; Fling et al., 2013), the model should simulate accurately the phenomenon of beta ERD/ERS generation and its dependency on TC. A specific protocol for the validation of the proposed model should include the recording of both:

- EEG signals from the SMA, during resting state condition and motor imagery tasks. The PSD and ERD/ERS should be extracted and fitted with model outputs to identify a *K*-value for each participant.
- Images of structural data using DTI for *in-vivo* scanning of human brain anatomy connectivity. The Functional Anisotropy could be extracted as indices of the TC (Halder et al., 2013).

The analysis of the correlation between K and Function Anisotropy could provide the validation of the model, demonstrating the dependency of the ERD/ERS amplitude on TC and the phenomenon of Transcallosal Inhibition. This is planned in future works.

## Conclusions

A two columns NMM was implemented to simulate (i) the right and left SMA and (ii) the mechanism of generation and propagation of beta ERD/ERS induced by a modulating input which simulates a motor imagery task. The model assumes that the way in which the input is transmitted is inhibitory, i.e., the inhibition of one cortex leads to the excitation of the other cortex and vice versa. The results can explain the ERD and ERS observed during the task and enforces the theory of inhibition, which supports that the two hemispheres are in constant mutually inhibitory relationship with each other and this competition is mediated by the corpus callosum.

## Author contributions

AM contributed to the design and conceptualization of the study, carried out the computational modeling, contributed to interpretation of the data and drafting of the manuscript. MU contributed to interpretation of the data and drafting of the manuscript. ML contributed to the design and conceptualization of the study and drafting of the manuscript. AC contributed to the design and conceptualization of the study and drafting of the manuscript.

### Conflict of interest statement

The authors declare that the research was conducted in the absence of any commercial or financial relationships that could be construed as a potential conflict of interest.
